# A Fiji protocol for analyzing puncta is a robust tool for measuring GLR-1::GFP accumulation in the ventral nerve cord of *C. elegans*

**DOI:** 10.17912/micropub.biology.001004

**Published:** 2023-12-14

**Authors:** Heino Hulsey-Vincent, Anna Athanasopoulos, Annette McGehee, Jennifer R. Kowalski, Caroline Dahlberg

**Affiliations:** 1 Biology, Western Washington University, Bellingham, Washington, United States; 2 Biology, Suffolk University, Boston, Massachusetts, United States; 3 Biological Sciences, Butler University, Indianapolis, Indiana, United States

## Abstract

In *C. elegans,* DAF-7/TGF-beta signaling regulates development, metabolism, and behavior. In addition loss of *daf-7* leads to an increase of the glutamate receptor GLR-1. In *daf-7(e1372)* mutants, GLR-1 tagged with GFP (GLR-1::GFP) accumulates in wide puncta along the ventral nerve cord of the animal. Previous automated analyses of GLR-1::GFP accumulation relied on the proprietary software, IgorPro, for measurement of GLR-1::GFP puncta size, intensity, and density. We did a side-by-side comparison of analyses by IgorPro and an open source macro written for Fiji to analyze images from animals expressing GLR-1::GFP in wild type and *daf-7(e1372)* backgrounds. Analyses by the two programs were in strong agreement and are in accordance with previously published data on the effects of *daf-7(e1372)* on GLR-1::GFP accumulation. Based on these data, we conclude that the Fiji platform is a robust method for analyzing the accumulation of a fluorescently-tagged neurotransmitter receptor and that the Fiji puncta plugin will be applicable for image analysis for other neural markers.

**Figure 1. Comparison of quantification of GLR-1::GFP puncta using Igor and Fiji software:**
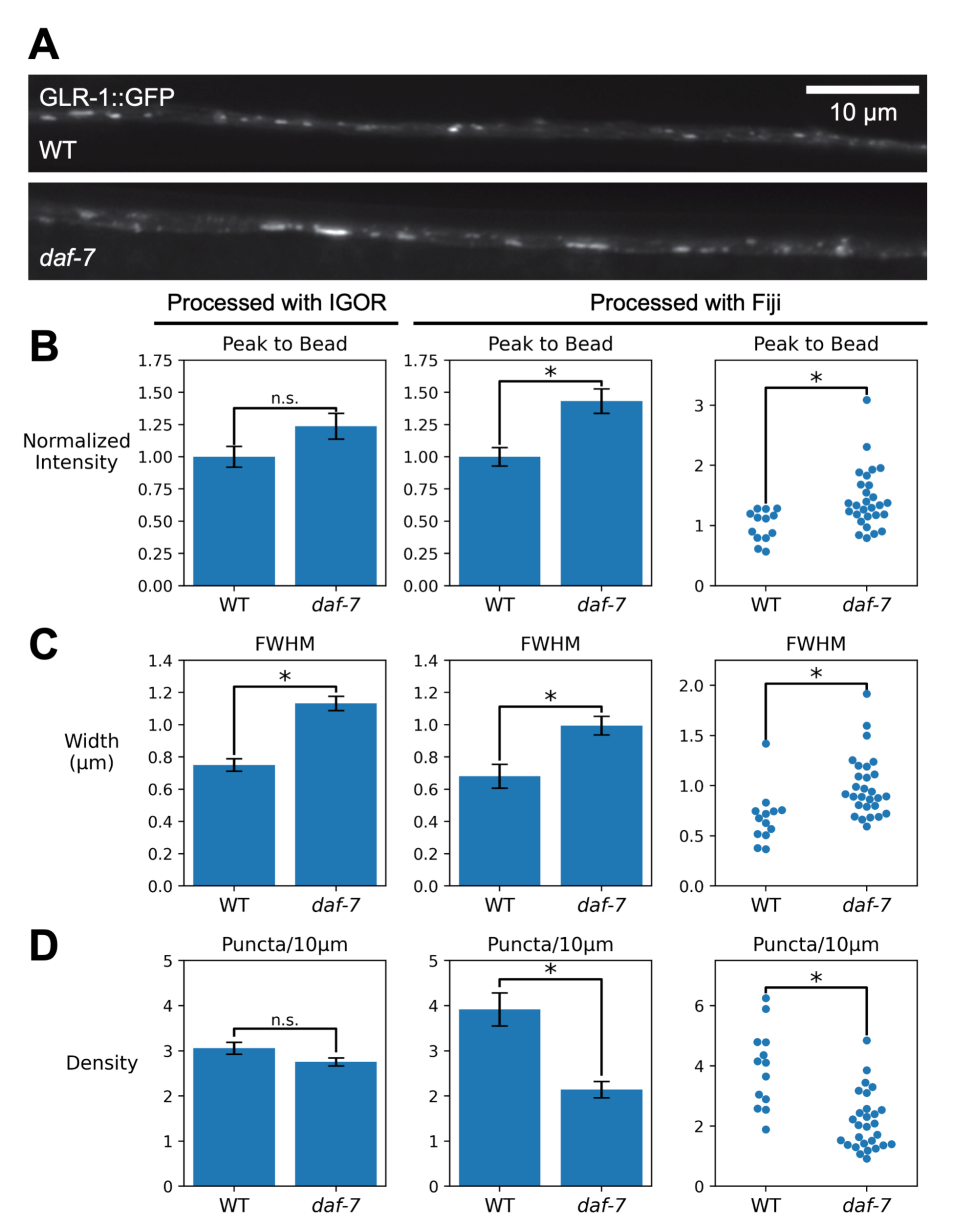
**A.** Representative images of WT and daf-7 mutant animals expressing GLR-1::GFP under the control of the *glr-1* promoter. **B-D.** Left, original quantification using IgorPro software; Center; analysis of the same data set using the Fiji macros. Error bars represent SEM. Right, ImageJ quantification displayed as swarm plots to show average intensity values for individual animals. **B.** Quantification of puncta intensity (peak-to-bead) per animal. **C.** Quantification of puncta width measured in microns as Full-width at Half-maximal (FWHM) fluorescence. **D** . Quantification of puncta density per 10 microns, by animal.

## Description

The *C. elegans* DAF-7/TGF-beta signaling pathway relays information about an animal’s environmental state to tissues throughout the body. Signaling through this pathway regulates the developmental decision to enter dauer (Golden and Riddle 1984, Thomas et al. 1993) , and acts in later developmental stages to regulate several aspects of metabolism, reproduction and behavior (Guimenny and Savage-Dunn 2013; Meisel et al. 2014; McKnight et al. 2014; Entchev et al. 2015; Fletcher and Kim 2017; Hilbert and Kim 2017; Pekar et al. 2017; Wexler et al. 2020; Schiffer et al. 2020; Tataridas-Pallas et al. 2021; Yamamoto and Savage-Dunn 2023). Additionally, DAF-7/TGF-beta pathway has previously been shown to regulate levels of the glutamate receptor GLR-1 (McGehee et al. 2015; McGehee 2019) .

Regulation of protein levels and localization within the nervous system of *C. elegans* can be studied by microscopy of fluorescently tagged proteins. The images produced require software to extract information, and return quantitative data. In previous studies, GFP-tagged GLR-1 (GLR-1::GFP) has been analyzed in the ventral nerve cord using the proprietary software, IgorPro. Those studies showed that animals harboring the *daf-7(e1372)* mutation have increased levels of GLR-1::GFP, which can be seen as both an increase in the fluorescence intensity of GLR-1::GFP puncta and an increase in the width of the puncta (McGehee et al. 2015, McGehee 2019) .

Here, we compared the quantification and analysis done using IgorPro with that using a new Fiji macro set (Hulsey-Vincent, et al., A 2023, Schindelin et al., 2012) . We analyzed the ventral nerve cord expression of GLR-1::GFP in *daf-7* mutants (N = 28) compared to wildtype animals (N = 13). Analysis using IgorPro and Fiji showed a 23% and 43% increase in peak-to-bead intensity values, respectively, with only the Fiji analysis having statistical significance ( Figure 1B, K -S test, p=0.57, Igor Pro, and p= 0.0028, Fiji),. Both IgorPro and the Fiji program similarly described 51% and 46% increases, respectively, in the width of GLR-1::GFP puncta in *daf-7* mutants compared with wild type ( Figure 1C, K -S test, p= 7.6e-6, Igor Pro, and p = 0.00076, Fiji). Analysis by IgorPro and Fiji showed a 10% and 45% decrease in puncta density ( Figure 1C, K -S test p=0.10, IgorPro, and p=0.0058, Fiji). We note that there was a difference in the quantification of peak-to-bead fluorescence and density measurements reported by the two analysis tools. For both peak-to-bead fluorescence and density, we see that the two analyses show similar trends. However, we are not able to determine the precise reason for the disparities in density measurements. In the future, it would be useful to do a careful investigation of the correlation between width and density measurements in mutants such as these. In addition, identification and quantification of puncta widths was reliable using the new Fiji protocol. This is important because “out-of-the-box” thresholding methods that are available in Fiji were not able to reliably identify all puncta in this data set.

Overall, we believe that the Fiji program provides an accessible and robust alternative to the Igor proprietary software package for quantification of GLR-1::GFP. Of major importance for this dataset was the consistency in accurately reporting the width of puncta, especially given the clear differences in the width of GLR-1::GFP puncta in *daf-7* mutant animals that have been previously reported (McGehee, et al., 2015; McGehee, 2019) . While we report differences in the change in puncta intensity using the open-sources Fiji macro compared with Igor, the overall trend from the analyses of the two software platforms is the same. The analysis reported here, in combination with analyses of GFP::SNB-1 images in the dorsal nerve cord motor neurons (Hulsey-Vincent, et al., A, B, 2023 ) suggests that the open-source Fiji puncta macro is useful for analyzing other accumulation of fluorescent proteins (neurotransmitter receptors, synaptic markers, etc.) in *C. elegans* neurons.

## Methods

Imaging was performed as previously described (McGehee et al., 2015) . Briefly, L4 worms with GLR-1:: GFP were paralyzed with 30 mg/ml 2,3-butandione monoxamine. Immobilized worms were placed onto a slide to image the anterior ventral nerve cord (VNC). Z-series stacks were collected using a Carl Zeiss Axiovert M1 microscope with GFP filter. Images of the VNC were collected with an Orca-ER CCD camera (Hamamatsu) and Metamorph (version 7.1) software (Molecular Devices). For the quantitative analyses of fluorescent puncta, the maximum intensity projections of Z-series stacks were utilized. Line scans of the ventral cord puncta were produced using the Meta-Morph (version 6.0), and analyzed with the Igor Pro (version 5) (Wavemetrics) (Burbea et al., 2002) . Statistical significance of any differences between wild type and mutant strain values was determined in Igor using a Kolmogorov–Smirnov Test. Graphs of puncta intensities show data normalized to wild type values.

Fluorescent intensity of 0.5 µm FluoSphere beads (Invitrogen) was measured to monitor the Arc lamp output.

Analysis with Fiji was performed as in Hulsey-Vincent, et al., A 2023 with the following settings: minimum puncta size = 10, radius = 2, sigma = 0.5, method = Phansalkar.

The data was tested for normality using a Shaprio-Wilks test, and was found to not be normally distributed (p < 0.05). To account for this, all data was tested for significant differences using a Kolmogorov–Smirnov Test.

## Reagents

**Table:** 

Strain name	Genotype	Available from the CGC
KP1147	*nuIs24(Pglr-1::glr-1::gfp)IV*	No (Rongo et al. 1998)
KP3079	*daf-7(e1372)III; nuIs24(Pglr-1:glr-1::gfp)IV*	No (McGehee et al. 2015)
